# Stable fly outbreaks in Brazil: a 50-year (1971-2020) retrospective

**DOI:** 10.1590/S1984-29612023017

**Published:** 2023-04-03

**Authors:** Antonio Thadeu Medeiros de Barros, Fernanda Galiano Soares, Thiago Nascimento de Barros, Paulo Henrique Duarte Cançado

**Affiliations:** 1 Embrapa Gado de Corte, Campo Grande, MS, Brasil,; 2 Universidade Católica Dom Bosco - UCDB, Campo Grande, MS, Brasil; 3 Universidade Estadual Paulista Júlio de Mesquita Filho - UNESP, Jaboticabal, SP, Brasil

**Keywords:** Stomoxys calcitrans, population explosions, livestock pest, Stomoxys calcitrans, explosões populacionais, praga pecuária

## Abstract

Over the last decades, stable fly (*Stomoxys calcitrans*) outbreaks associated with agricultural and/or livestock production systems have become a serious problem in some Brazilian locations. This article presents a survey on the history, evolution and mapping of such outbreaks in Brazil over five decades (1971-2020). Outbreaks (n= 579) were recorded in 285 municipalities from 14 states, mainly associated with by-products from the ethanol industry (82.7%), *in natura* organic fertilizers (12.6%) and integrated crop-livestock systems (3.1%). Few cases were reported until the mid-2000s, progressively increasing since then. Outbreaks associated with ethanol mills occurred in 224 municipalities, mainly in Southeast and Midwest states, while those associated with organic fertilizers (mainly poultry litter and coffee mulch) affected 39 municipalities, mostly in the Northeast and Southeast states. More recently, outbreaks in integrated crop-livestock systems during the rainy season have occurred in Midwest states. This survey highlights the magnitude of the problem of stable fly outbreaks in Brazil and its relationship with environmental public policies, agricultural production chains and regional trends. Specific public actions and policies are urgently needed to prevent their occurrence and impact in the affected regions.

## Resumo

Nas últimas décadas, surtos da mosca-dos-estábulos (*Stomoxys calcitrans*), associados a sistemas de produção agrícola e/ou pecuária, tornaram-se um grave problema em algumas localidades brasileiras. Este artigo apresenta um levantamento sobre a história, evolução e mapeamento desses surtos no Brasil ao longo de cinco décadas (1971-2020). Surtos (n= 579) foram registrados em 285 municípios de 14 estados, principalmente associados a subprodutos da indústria do etanol (82,7%), fertilizantes orgânicos *in natura* (12,6%) e sistemas de integração lavoura-pecuária (3,1%). Poucos casos foram registrados até meados dos anos 2000, aumentando progressivamente desde então. Surtos associados a usinas de etanol ocorreram em 224 municípios, principalmente no Sudeste e Centro-Oeste, enquanto surtos associados a fertilizantes orgânicos (principalmente esterco aviário e palha de café) afetaram 39 municípios, principalmente nos estados do Nordeste e Sudeste. Mais recentemente, surtos em sistemas de integração lavoura-pecuária, durante a estação chuvosa, têm ocorrido no Centro-Oeste. Este levantamento ressalta a magnitude do problema dos surtos da mosca-dos-estábulos no Brasil e sua relação com políticas públicas ambientais, cadeias produtivas agrícolas e tendências regionais. Ações e políticas públicas específicas são urgentemente necessárias para prevenir sua ocorrência e impacto nas regiões afetadas.

## Introduction and General Aspects

*Stomoxys calcitrans* (Linnaeus, 1758) is a cosmopolitan species, known worldwide as stable fly. Hematophagous and opportunistic, these flies primarily attack domestic (Foil & Hogsette, 1994) and wild (Fosbrooke, 1963) mammals, but occasionally, also birds (Gonçalves & Veiga, 1998) and humans (Hogsette et al., 1987). Their painful bites result in behavioral changes (Mullens et al., 2006) and significant production losses. Economic losses to the livestock industry in Brazil are estimated at US$ 335 million per year (Grisi et al., 2014), excluding those from outbreaks. In the United States, yearly production losses exceed US$ 2 billion (Taylor et al., 2012).

Stable fly reproduction is closely associated with decaying plant matter and its larvae develop in various substrates (Corrêa et al., 2013; Cook et al., 2018). Fresh bovine fecal masses on pastures are not a good substrate for larval development, however, cattle manure and urine mixed with cattle feed residues and other organic material provide suitable conditions for the abundant development of stable flies (Hogsette et al., 1987; Broce et al., 2005; Cook et al., 2018). Such situations allow the maintenance of local fly populations, occasionally leading to higher infestations in livestock operations.

Influenced by environmental conditions, the abundance of stable flies fluctuates throughout the year; however, in situations influenced by agricultural practices, management may play the most important role (Dominghetti, 2017). Natural stable fly populations in Brazil have shown a single yearly population peak in November or December (Bittencourt & Moya-Borja, 2000; Rodríguez-Batista et al., 2005) or two peaks during the rainy season, with a lower peak in March in southern Brazil (Rodríguez-Batista et al., 2005). Under natural conditions, the beginning of the rainy season during the spring stands out as the period of highest abundance of this fly in the country.

Regardless of natural population peaks, the occurrence of alarming infestations have been frequent at different times of the year, affecting production and animal welfare in several locations (Dominghetti et al., 2015). Those episodes are commonly called “outbreaks” and, for the purpose of this review, an outbreak is a sudden increase in the stable fly population associated with their massive production in organic residues/by-products from agricultural, agro-industrial and/or livestock production systems, affecting animals and humans. Similar situations have been reported abroad as well (Martínez Perilla et al., 2013; Solórzano et al., 2015; Cook et al., 2018) and illustrate not only the wide variety of developmental substrates used by stable flies, but also the scope and diversity of agricultural production systems involved in outbreak episodes.

Given the adaptability of this parasite to such a variety of environments, there is potential risk for stable fly outbreaks in several livestock regions of Brazil, as the production systems associated with outbreaks elsewhere are also present in the country. In the vast majority of cases, stable fly outbreaks result from the large production and/or improper use or management of organic substrates (feed, fertilizers, etc.), residues and byproducts in agricultural and livestock production systems (Skoda et al., 1991; Broce et al., 2005; Solórzano, 2014; Cook et al., 2018). Such substrates provide favorable conditions for the large-scale proliferation of those flies and the subsequent dispersal of host seeking adults to livestock facilities, as well as to peri-urban and recreational areas (King & Lenert, 1936; Cook et al., 1999).

Natural high infestations tend to show a smaller scope, lower intensity and shorter duration than outbreaks. Fewer than 30 stable flies per host are normally observed for a few weeks during natural infestation peaks (Rodríguez-Batista et al., 2005; Zimmer et al., 2010). However, during outbreak infestations hundreds of flies per animal can occur for up to several months (Herrero et al., 1989; Solórzano, 2014). Confined animal facilities are particularly favorable for large-scale organic waste production; consequently, eventual failures in residue management lead to foci of stable flies developing in considerable numbers (Meyer & Petersen, 1983; Thomas et al., 1996), affecting animals on the source farm and in the neighborhood.

While natural population peaks are determined by favorable weather conditions and tend to occur shortly after the onset of rains, outbreaks can occur at any time, directly related to management practices and the abundance of available developmental sites (Dominghetti, 2017). As informed by cattle producers, the alarming intensity and duration of stable fly attacks, has led to changes in livestock management, including the movement of animals to unaffected areas, changes in the milking schedule, early sale of animals and, eventually, the abandonment of the livestock production in affected areas.

In Brazil, outbreaks of *S. calcitrans* associated with agricultural activities date back to the 1970s (Nakano et al., 1973). Since then, the expansion of different production chains, changes in management, and regulatory policies have deeply influenced the frequency and severity of outbreaks in the country (Dominghetti et al., 2015).

Although *S. calcitrans* outbreaks are not uncommon in some regions of Brazil, records of such episodes in the scientific literature are relatively scarce. The dissemination of information through digital media is significantly greater, but it is dispersed and is usually only available on websites with a local or regional scope. The objective of this survey was to gather information on the occurrence of stable fly outbreaks in Brazil during a 50 year period (1971-1920) and to present an overview of their history, evolution and distribution throughout the country.

## Survey Methodology

This survey is the result of more than a decade of searching and cataloging records on the subject. During this period, all information on outbreaks obtained from extensive searches in the technical-scientific literature and internet, consultations and reports from professionals of the agricultural sector, and information provided by producers and rural associations (personally or through telephone contacts, electronic mail and WhatsApp) were recorded.

Retrospective internet searches on the occurrence of stable fly outbreaks, as well as the organization of all the information obtained, have been conducted systematically since 2009. In more recent years, the searches have been intensified to expand and complement the information from previous years.

Searches in digital media were conducted through Google using several keywords related to the subject and their combinations, allowing for a broader search. During surveys, it was noticed that some words present in the headlines of articles, although not directly related to the subject, resulted in the recovery of new records. All information from the media was saved in pdf format (portable document format) and organized in a database. Words (in Portuguese) used in this research included: attack, beronha, bironha, bovine, chicken litter, poultry manure, cattle, fly, sugarcane fly, mill fly, stillage fly, manure, bagasse fly, cattle fly, stable fly, concern, injury, problem, producer, herd, *Stomoxys calcitrans*, outbreak, sugarcane mill and vinasse. Hyphenated words were queried both with and without hyphen.

Extensive surveys on stable fly outbreaks in São Paulo state were performed in 2016, 2018 and 2019 by the São Paulo Department of Agriculture and Supply (SAA-SP), through its Coordination of Sustainable Rural Development (CDRS - previously CATI). Such surveys were essential to expand, and complement, the extensive list of municipalities affected by stable fly outbreaks in the state of São Paulo.

Much information, complaints and reports on the occurrence of outbreaks were communicated by professionals from the agricultural sector, ranchers, and technicians from affected livestock properties through personal contacts as well as the aforementioned means of communication.

The species of the livestock pest causing the outbreaks was not identified by specialists in most cases. However, its morphological, biological and behavioral characteristics (mainly hematophagy), as well as the epidemiology of the outbreaks, associated with agricultural production systems, allow to conclude that it was the stable fly. Photos and videos of flies and outbreaks were sometimes requested by the authors to confirm previous field identification of the species by producers, veterinarians and technicians.

Based on outbreak origin, the records were classified as “sugarcane mills”, “fertilizers”, “integrated production systems” and “unknown”, and organized by the site and year of occurrence, facilitating subsequent checking and analysis. Multiple reports of the same outbreak were considered as a single record.

Despite the exhaustive effort, this survey obviously does not represent all cases of stable fly outbreaks occurring during five decades in Brazil. Limited information was available before popularization of the internet and, even more recently, outbreak cases certainly were not recorded due to the lack of disclosure. However, the collection of records gathered over the years covers the majority of information published or made available in the media in addition to the many personal reports. These reports represent a robust history of the occurrence, evolution and association of these events with agricultural and livestock production systems and with environmental policies, as well as their geographical distribution in different regions of the country.

## History and Evolution of Outbreaks in Brazil

A total of 579 records of stable fly outbreaks in Brazil were found for the 50-year period covered by this survey. The temporal evolution of those outbreaks is presented by decade, with yearly distribution for the last twenty years (Table 1), affecting 285 municipalities from 14 states (Table 2). In general, most outbreak records were obtained from the internet (62.5%), followed by personal communications (31.8%) and technical-scientific publications (5.7%). Specifically for the outbreaks associated with sugarcane mills, these sources contributed with 63.4%, 29.9% and 6.7% of the records, while for the outbreaks from other causes they were 58.4%, 40.6% and 1.0%, respectively.

**Table 1:** Absolute frequency of stable fly outbreak records in Brazil, by origin, over five decades (1971 to 2020).

	Mills	Fertilizers	ICLS	Unknown	Total
1971-1980	2	2	0	0	4
1981-1990	0	0	0	0	0
1991-2000	0	4	0	0	4
2001-2010	40	9	0	1	50
2001	0	0	0	0	0
2002	1	0	0	0	1
2003	1	4	0	0	5
2004	0	0	0	0	0
2005	1	0	0	0	1
2006	0	0	0	0	0
2007	3	0	0	0	3
2008	7	1	0	0	8
2009	10	2	0	0	12
2010	17	2	0	1	20
2011-2020	437	58	18	8	521
2011	15	1	1	0	17
2012	17	3	0	0	20
2013	16	8	0	1	25
2014	19	1	1	1	22
2015	33	5	0	1	39
2016^*^	106	3	1	1	111
2017	36	3	2	1	42
2018*	107	14	3	2	126
2019*	76	6	6	1	89
2020	12	14	4	0	30
Total	479	73	18	9	579

Mills - sugarcane mill for ethanol production; ICLS - integrated crop-livestock systems.

* Stable fly outbreak survey conducted by CATI/CDRS in São Paulo.

**Table 2:** Relative frequency (%) of stable fly outbreak records by origin in Brazil from 1971 to 2020.

Region/State	Mills	Fertilizers	ICLS	Unknown
**North**	**0.0**	**0.0**	**0.0**	**100.0**
Pará	0.0	0.0	0.0	100.0
Tocantins	0.0	0.0	0.0	100.0
**Northeast**	**0.0**	**94.4**	**3.7**	**1.9**
Bahia	0.0	88.9	7.4	3.7
Maranhão	0.0	100.0	0.0	0.0
Pernambuco	0.0	100.0	0.0	0.0
**Midwest**	**83.9**	**0.0**	**14.3**	**1.8**
Goiás	85.7	0.0	14.3	0.0
Mato Grosso do Sul	92.4	0.0	7.6	0.0
Mato Grosso	50.0	0.0	38.9	11.1
**Southeast**	**94.6**	**4.9**	**0.0**	**0.5**
Espírito Santo	18.8	81.3	0.0	0.0
Minas Gerais	87.5	0.0	0.0	12.5
Rio de Janeiro	0.0	100.0	0.0	0.0
São Paulo	98.4	1.3	0.0	0.3
**South**	**0.0**	**66.7**	**0.0**	**33.3**
Santa Catarina	0.0	100.0	0.0	0.0
Rio Grande do Sul	0.0	50.0	0.0	50.0

Mills - sugarcane mill for ethanol production; ICLS - integrated crop-livestock systems.

Such events were associated with different agricultural production systems, but most were associated with the sugarcane industry. Records of stable fly outbreaks associated with ethanol mills totaled 479 (82.7%), affecting 224 municipalities in six states. Outbreaks associated with activities other than ethanol production totaled 100 cases (17.3%) in 61 municipalities and 14 states. Organic fertilizers were associated with 12.6% (n= 73) of the outbreak records and 3.1% (n= 18) occurred in integrated crop-livestock systems. For a small number of cases 1.6% (n= 9) the cause was unknown.

The relative scarcity of records prior to 2008 can be explained by an apparent lower frequency of outbreaks at that time as well as the still incipient growth of the internet in the country. According to De Bettio (2015), only 17% of urban households in Brazil had a computer in 2005 and just 39% in 2010, with an even lower proportion of those connected to the internet.

Until the mid-2000s, records of stable fly outbreaks were sporadic and generally associated with the use of organic fertilizers. Although disclosure limitations obviously reduced the number of records available for the period, the rare cases reported and the much lower agricultural production (mainly grains and sugarcane) compared with current levels, indicate that stable fly outbreaks were not a major problem at that time. The state of Espírito Santo can be considered an exception, having a few municipalities affected by stable fly outbreaks associated with the use of coffee processing residues as organic fertilizers.

In the following decades, a marked change took place regarding both the origin and dynamics of stable fly outbreaks throughout the country, largely dominated by the expansion of sugarcane and associated ethanol production. For a broad understanding of the evolution of stable fly outbreaks in the country, a retrospective analysis of the predisposing and determining factors that led to the current situation is provided.

Several decomposing plant substrates are attractive to stable flies for oviposition and larval development. Once the necessary conditions for an outbreak are present, its intensity depends on the quantity and quality of substrates, while its duration depends on the continuity of the substrate supply. Of course, abiotic factors, such as weather conditions and local management practices adopted contribute to the severity as well. Regarding their origin and despite the great diversity of potential developmental habitats, stable fly outbreaks were classified into three major categories: a) Agricultural wastes, residues and by-products, b) Organic fertilizers, and c) Livestock and integrated crop-livestock systems.

## Outbreaks Associated with Agricultural Wastes, Residues and By-products

This category includes outbreaks associated with the massive proliferation of stable flies in several substrates generated by agricultural or agro-industrial production systems. This situation has been observed with pineapple production in Costa Rica (Solórzano, 2014; Solórzano et al., 2013, 2015), palm oil in Colombia (Mora et al., 1997; Garbiras et al., 1997; Martínez Perilla et al., 2013) and Costa Rica (Vargas, 2020), and vegetables in Australia (Cook et al., 1999, 2011, 2018). Although no records of stable fly outbreaks arising from those specific substrates have been reported to date in Brazil, it is important to keep in mind that these production systems are present in the country and the stable fly is able to adapt to different environments with decaying plant material.

In Brazil, outbreaks occurring in residues and organic by-products generated in ethanol mills are mainly associated with sugarcane mulch (layer of leaves and tips of sugarcane stalks left on the ground during mechanized harvesting), vinasse (liquid remaining after the distillation of fermented sugarcane juice), filter cake (sediment from the filtration of sugarcane juice) and vinasse sludge (decanted vinasse sediment accumulated in tanks and canals during the harvest season). Vinasse is one of the main by-products generated by the ethanol production system (10 to 18 liters per liter of ethanol); rich in organic matter and nutrients, vinasse is applied on sugarcane fields as a fertilizer (Silva et al., 2007). Although stable flies are able to develop in sugarcane mulch and bagasse (fibrous material resulting from sugarcane milling), the main substrates for larvae development are filter cake and sugarcane mulch mixed with vinasse (Corrêa et al., 2013). Furthermore, the vinasse sludge promotes massive production of stable flies in a relatively small area and its careless disposal after reservoirs and canals are cleaned may cause unexpected outbreaks even during the off-season.

The association between sugarcane mills and the massive production of *S. calcitrans* has been clearly demonstrated, as a consequence of the availability and suitability of organic substrates resulting from the ethanol production process (Bittencourt, 2012; Corrêa et al., 2013; Reis e Silva et al., 2013; Dominghetti et al., 2015; Souza et al., 2021). The capacity of stable fly production in organic by-products of sugarcane mills is considerably high. Reis e Silva et al. (2013), extrapolating results from laboratory studies, estimated a production of 1,000 to 13,000 flies per ton of sugarcane mulch plus vinasse, resulting in 10,000 to 130,000 flies/hectare. Using emergence traps placed on different substrates at a sugarcane mill, Corrêa et al. (2013) obtained an average monthly production of about 24 flies/m^2^ in mulch-vinasse and 56 flies/m^2^ in filter cake, representing 242,000 to 558,000 flies/ha, respectively. Considering the respective areas covered by each substrate at the mill under study, the authors estimated an average monthly production of 37,000 stable flies from the filter cake and 24 million flies in mulch-vinasse.

The proximity between feedlots and sugarcane mills, with their extensive areas of vinasse application, pose a risk of a large-scale stable fly development and the subsequent outbreaks. Such closeness between livestock and ethanol mills facilitates the movement of flies between development and feeding sites, which favors rapid population growth. Thus, even a small population of stable flies maintained at cattle ranches and feedlots during the sugarcane off-season will allow a considerable influx of flies to the sugarcane fields as soon as the harvest resumes and the sweet odor of residues and by-products is dispersed. In such a situation, the occurrence of an outbreak is a matter of time and subsequent generations will increase exponentially taking advantage of the increasing availability of developmental sites.

### Chronological recording

The first record of a stable fly outbreak associated with residues from sugarcane mills in Brazil dates back to 1971, when a “huge population”, causing problems for animals and rural workers, developed in vinasse discharge areas of a sugarcane mill in Piracicaba, SP (Nakano et al., 1973). Later in that decade, an outbreak associated with a sugarcane mill occurred in the municipality of Boa Esperança, Espírito Santo state (C. Fanton, personal communication).

No records of outbreaks of stable flies due to sugarcane activities were found for the 1980s and 1990s. However, a large increase in the abundance of houseflies (*Musca domestica*) was registered in two municipalities in São Paulo state, related to the application of vinasse to sugarcane fields (Buralli & Guimarães, 1985) and use of poultry litter as a fertilizer (Buralli et al., 1987).

The following decade (2000s) was a transition period, with a relatively discrete, but progressive, increase in the occurrences of outbreaks in its final half (Table 1), mostly associated with sugarcane mill activities. In the first half of that period, a few outbreaks were reported, including an episode in the state of Espírito Santo caused by vinasse sludge accumulated in the bottom of canals and in disposal areas (C. Fanton, personal communication), a case in the state of Mato Grosso where vinasse application to the fields led to an explosion of the stable fly populations seriously affecting nearby livestock, and a similar situation in 2005 in the state of São Paulo (R. A. Gomes, personal communication).

In 2007, three outbreaks associated with sugarcane mills were reported in the state of São Paulo. In the municipality of União Paulista, stable fly proliferation in filter cake and sugarcane mulch plus vinasse reduced cattle weight by about 50 kg and milk production by 15% to 40% (Gomes, 2009). In 2008, a stable fly outbreak was reported for the first time in the state of Mato Grosso do Sul (Barros et al., 2010).

In the following year (2009), stable fly outbreaks associated with sugarcane mills continued to occur in the states of São Paulo (5), Mato Grosso do Sul (4) (Koller et al., 2009; Barros et al., 2010), and for the first time in Minas Gerais (Barros et al., 2010). In 2010, such outbreaks continued in São Paulo (Oda & Arantes, 2010) and Mato Grosso do Sul (Kassab et al., 2012). An outbreak in the state of Mato Grosso due to a large proliferation of stable flies in vinasse residues deposited in canals, caused serious damage to production and death of animals, resulting in civil actions against the sugarcane mill (Mato Grosso, 2010). The first stable fly outbreak was reported in Goiás (Alves, 2012).

Stable fly outbreaks associated with sugarcane activities related to ethanol production increased in alarming proportions in the period of 2011-2020, an increase above 1,000% in relation to the previous decade, as a consequence of the expansion of production areas, increase in scale of production, changes in waste and by-product management, and adaptation of the fly to new environments.

Until the middle of the decade (2010-2014) the yearly number of outbreaks related to the ethanol industry ranged from 15 to 19 in the affected states of Espírito Santo, Goiás, Mato Grosso do Sul, Mato Grosso and São Paulo. A noticeable increase in the number of reports occurred from 2015 onwards, reaching 107 municipalities in 2018, the year with the highest frequency (Table 1). The increase in the number of cases in the second half of that decade was greatly influenced by the extensive surveys carried out in 2016, 2018 and 2019 by CDRS/CATI and its regional offices on the occurrence of stable fly outbreaks in SP. The highest number of outbreaks coinciding with the three years of the CDRS/CATI surveys shows that records from other years most likely underestimated the frequency of ethanol-related outbreaks in that state, especially in the last decade. However, it is worth mentioning that the frequency of outbreaks increased in those years regardless of the additional information from the CDRS/CATI surveys, with the outbreak frequencies oscillating among years according to the preventive measures adopted (or not) in each site and the disclosure (or not) of the cases by local media.

### Contextualized retrospective analysis (1971-2020)

In 1978, driven by issues related to the quality of inland waters and the ecological balance of inland aquatic environments, the disposal of vinasse in water bodies was prohibited in the country by the Portaria/GM 323, from November 29, 1978 (Brasil, 1978). This led to the application of vinasse in sugarcane fields, in a process known as “fertigation”. In this practice, the vinasse is applied over the area by a sprinkler system with a hose driven cannon (“hydro roll”). At that time, fertigation was carried out after harvesting of the burned sugarcane, thus resulting in the application of vinasse on bare or ash covered ground and its quick infiltration into the soil. Regardless of other environmental issues, the use of this management practice for more than 20 years (since its beginning until 2001) left no records of stable fly outbreaks associated with the ethanol industry (Table 1).

However, this situation changed radically after the Presidential Decree 2,661 from July 08, 1998 (Brasil, 1998), which, for environmental and public health reasons, established norms regarding the use of fire in agricultural and forestry practices, mandating the gradual elimination of fire as a method of scavenging sugarcane over a period of 20 years. Later, the State Law 11,241 from September 19, 2002 (São Paulo, 2002) established a specific schedule for the state of São Paulo, with complete elimination of burning in both mechanized (20 years) and non-mechanized (30 years) harvest areas. These regulations set the stage for a gradual process of mechanization of sugarcane harvesting, reducing by at least 25% the burned area every five years.

**Figure 1:**
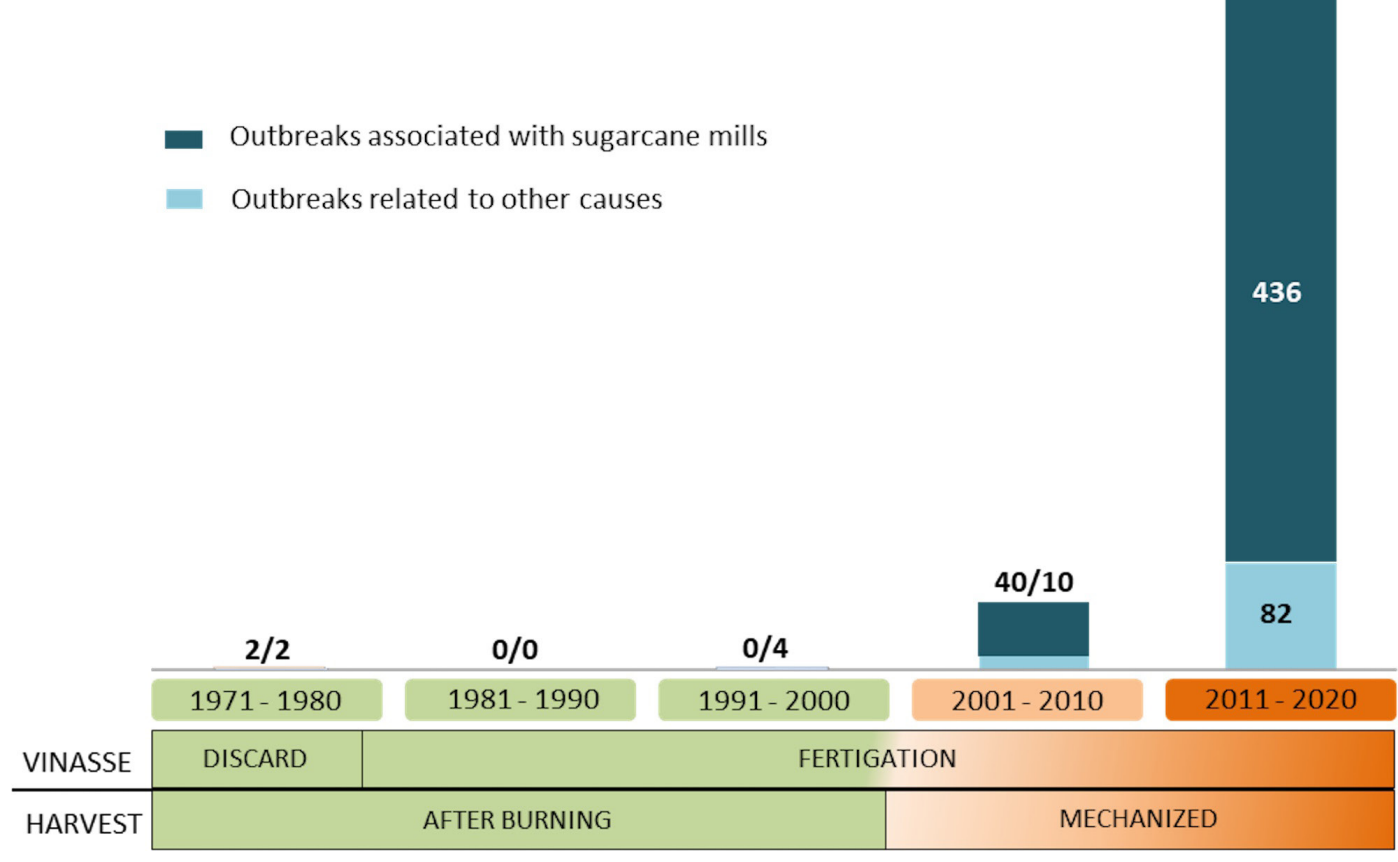
Timeline of the most important changes in sugarcane management practices related to stable fly outbreaks over 50 years (1971-2020) in Brazil.

Burning before manual harvest eliminated sugarcane leaves and left little residue on the ground, thus facilitating and enhancing infiltration of the vinasse applied after harvest. However, in the mechanized harvesting, the leaves and the tips are cut and thrown on the ground, creating a cover layer of mulch (Oliveira et al., 1999). The deposition of the leaf biomass on the ground is aggravated by the accumulation of mulch resulting from successive harvests. This change in sugarcane management had profound consequences in relation to the proliferation of stable flies and the occurrence of outbreaks associated with this production system (Figure 1). Such situation was aggravated by the expansion of the bioenergy sector and the construction of sugarcane mills in traditional livestock production areas (Barros et al., 2010).

From then on, the scenario of outbreaks of the stable fly in the country was gradually but deeply changed. Previously sporadic (in most cases), and usually related to the use of organic fertilizers, outbreaks associated with sugarcane mills increased in frequency, duration and intensity with serious socioeconomic consequences. In the producer's view, stable fly outbreaks become the main problem of the property in the affected localities.

The end of the first five-year period established by the São Paulo State Law 11,241 (São Paulo, 2002) for the gradual elimination of fire started a progressive increase in the number of stable fly outbreaks associated with ethanol mills in the state. Although on a smaller scale (because of the smaller number of mills), a similar situation occurred in other states. In 2007, a protocol of intent signed between the Secretary of the Environment of the State of São Paulo and the União da Indústria de Cana-de-Açúcar (UNICA) brought about the banning of burning in mechanized areas in 2014 and its complete elimination by 2017 (Ronquim, 2010). Compliance with this schedule coincided with a sudden increase in the number of outbreaks starting in 2015.

Although the ban of vinasse disposal in water bodies and the consequent fertigation of sugarcane fields did not influence the occurrence of stable fly outbreaks in the following 20 years, the prohibition of burning deeply changed this situation, an issue that persists. In addition to the consequences resulting from fertigation with vinasse on mechanized harvesting fields, the amplification of outbreaks associated with sugarcane mills was also influenced by the expansion of the bioenergy sector in the country, driven by the National Alcohol Program (Proálcool) and fuel marketing.

In this context, increasing demand for ethanol with the introduction of “flex-fuel” (alcohol and gasoline) cars in the Brazilian market in 2003 led to a 73% expansion of the sugarcane cultivated area in the following five years (2003-2008) in São Paulo state (Rudorff et al., 2010). The number of municipalities in that state affected by outbreaks doubled in 2008 compared with the previous year, coinciding with a 50% reduction in the area of sugarcane burned prior to harvest in the state and the expansion of the sugarcane cultivated area (Rudorff et al., 2010).

At the same time, the annual growth rate of ethanol production between 2003/2004 and 2013/2014 in the Midwest region of Brazil was more than twice the national average due to the increase in domestic consumption with the massive production of “flex-fuel” cars (Barbosa et al., 2020). The expansion of the sugar-alcohol sector toward the Midwest in 2006/2007 and 2007/2008 harvests (Rudorff et al., 2010), not only coincided with the first outbreaks recorded shortly afterwards in Mato Grosso do Sul state in 2008 and 2009 (Koller et al., 2009; Barros et al., 2010) and in Goiás state in 2010 (Alves, 2012), but with the beginning of a series of yearly outbreaks in the region since then (Kassab et al., 2012; Dominghetti et al., 2015; Dominghetti, 2017).

The evident rise in the number of stable fly outbreaks related to the sugarcane sector over the last decade is worrying. From the late 1970s to the late 1990s (period with fertigation after pre-harvest sugarcane burning) there were no report of outbreaks associated with ethanol mills; however, in the following decade (2001-2010) 40 outbreaks were observed and this number surpassed all other causes of outbreaks (n= 10). In the subsequent decade (2011-2020), 437 outbreaks associated with sugarcane mills were recorded, more than five times the sum of all other sources (n= 84) (Table 1). In that period, the number of outbreaks associated with sugarcane mills and other causes increased 1,093% and 840%, respectively, relative to the previous decade.

The association between the residues/by-products generated in sugarcane mill activities and the risks of stable fly outbreaks is currently well known by the bioenergy sector. The investment and the efficiency of the preventive measures adopted locally are variable, but, in general, the subject has been included in the agenda for discussion and priorities of several bioenergy companies. Although stable flies do not inflict damage on the sugarcane plant, they are pests of the Sugarcane and Bioenergy Sector, with several negative implications arising from the number and severity of the associated outbreaks, including the lawsuits and the negative media coverage. The most relevant negative aspects include legal issues such as Occurrence Bulletins and Conduct Adjustment Terms, threats to the credibility and image of the Sector, and considerable investments in preventive and corrective measures.

Gomes et al. (2018) estimated yearly economic losses of around BRL 3.5 million for an outbreak affecting 90 livestock properties near a sugarcane mill in the state of São Paulo. Based upon similar outbreaks associated with sugar and ethanol plants in the country in 2016 (n= 106), 2018 (n= 107) and 2019 (n= 76), years in which more complete and comprehensive surveys were carried out in that state (the one with the highest frequency of outbreaks), and extrapolating a loss of BRL 3.5 million/year/outbreak, an annual economic impact of BRL 262.5 to BRL 374.5 million can be attributed to outbreaks of this nature. In a preliminary analysis, Cançado (2020) estimated the annual economic losses caused by stable fly outbreaks at around US$ 108 million, which represents approximately BRL 568 million at the current exchange rate (US$ 1.00 = BRL 5.26). These estimates complement, and considerably expand, the damage caused by this parasite to livestock, previously estimated by Grisi et al. (2014) at US$ 335.46 million yearly, excluding outbreaks. It is important to emphasize that such losses do not take into account reproductive aspects, such as problems with estrus, libido, abortions and dystocic deliveries, or abandonment of newborns and injuries in calves as a consequence of herd crowding and irritation, common situations observed during outbreaks (Barros et al., 2010; Bernardes et al., 2018).

## Outbreaks Associated with Organic Fertilizers

Outbreaks associated with the massive development of this fly in organic fertilizers applied to crops have occurred in Brazil mainly due to the use of poultry litter, coffee and rice mulch. However, it is also suspected that the stable flies develop in fermenting accumulated post-harvest residues (mulch) of soybeans and peanuts. The improper use of organic fertilizers such as rice mulch and coffee processing residues has caused outbreaks of *S. calcitrans* in Costa Rica (E. Vargas, personal communication).

Stable fly outbreaks have been reported in several regions of the country due to the use of organic fertilizers in crops such as bananas, plantains, sweet potatoes, coffee, yams, ginger, vegetables, oranges and papayas, in addition to pastures. However, outbreaks involving organic fertilization are not limited to those crops. Less common situations involving fertilizers included the application of beer yeast in pastures (A. Bittencourt, personal communication), hog effluents, and storage of organic fertilizers by a producer company (Marvadeza, 2019).

### Chronological recording

Stable fly outbreaks associated with the use of organic fertilizers were first recorded in 1970s in two municipalities from the state of Espírito Santo. At least one was due to fertilization of coffee with coffee wastes (Matioli & Arleu, 1979). Based on subsequent outbreaks in the region, it is likely that the other outbreak had the same origin.

Few records of stable fly outbreaks are available from the 1980s and 1990s. Guimarães (1986) cited by Aguiar-Valgode & Milward-de-Azevedo (1992) reported intense stable fly infestations in the northeastern states of Paraíba and Rio Grande do Norte, and that the use of rice mulch in coffee plantations and poultry litter in sugarcane fields supported the development of immature stable flies in the states of Goiás, Minas Gerais, Paraná and São Paulo. Outbreaks in the state of Espírito Santo occurred also on banana plantations due to the use of coffee mulch as a fertilizer (EMCAPA, 1998).

At the beginning of the 2000s, sporadic outbreaks were reported in the state of Rio de Janeiro due to the fertilization of vegetables with poultry litter and use of brewing yeast on pastures (A. Bittencourt, personal communication). In the Espírito Santo state the intense proliferation of stable flies due to the improper use of organic fertilizers led to banning the use of decomposing substrates (such as coffee straw, poultry litter and leftover feed) as crop fertilizers by the Portaria n° 23-R from December 02, 2003. Even in this decade, reports of stable fly outbreaks began in some states of the northeastern region, such as Bahia, due to the use of poultry litter and coffee mulch as fertilizers for papaya, banana and coffee (Sulbahia News, 2009), and in Pernambuco, associated with the use poultry litter as a fertilizer for yams; the latter outbreak led to cattle deaths (Redação Nordeste Rural, 2015).

In the last decade (2011-2020), although less frequent, outbreaks unrelated to sugarcane activities have been reported in several states from the North (Pará and Tocantins), Northeast (Bahia, Maranhão and Pernambuco), Midwest (Goiás, Mato Grosso do Sul and Mato Grosso), Southeast (Espírito Santo, Minas Gerais and São Paulo) and South (Rio Grande do Sul and Santa Catarina) regions. For the most part, such outbreaks were associated with the use of organic fertilizers, particularly poultry litter (poultry manure mixed with sawdust) on various crops. Since this practice is seasonal, stable fly outbreaks tend to repeat annually at those sites. The occurrence of outbreaks associated with the use of organic fertilizers from 2011 to 2020 increased more than 500% relative to the previous decade.

In the Northeast region, outbreaks were reported in 28 municipalities during the 2010s, mostly associated with poultry litter. The most affected state was Bahia, with outbreaks in 15 municipalities associated with poultry litter and possibly coffee mulch in the cultivation of papaya, plantains and coffee (Mídia Bahia, 2019; Gomes, 2020). In the state of Pernambuco, stable fly outbreaks associated with poultry litter on yams have been reported between September and February in ten municipalities for several years (Redação Nordeste Rural, 2015; 2018; 2019). More recently, stable fly outbreaks in 2019 and 2020 associated with the use of poultry litter in banana plantations are under investigation by AGED - State Agency for Agricultural Defense of Maranhão (L. M. Costa Júnior, personal communication).

Relatively few stable fly outbreaks not associated with the sugarcane activity have been reported in southeast states, including Espírito Santo, Minas Gerais and São Paulo. Outbreaks have occurred in the state of Espírito Santo due to the use of poultry litter in papaya (C. Fanton, personal communication), ginger, sweet potato and yam crops, as well as the use of coffee mulch as an organic fertilizer. These outbreaks resulted in a public civil action (Tribunal de Justiça do Estado do Espírito Santo, 2016). Outbreaks occurred yearly in the state of Minas Gerais between December and February (rainy season) during the coffee harvest; however, the use of swine effluents as a pasture fertilizer may have contributed as well.

Although the vast majority of outbreaks in São Paulo state were directly related to the ethanol industry, a few cases can be attributed to pasture fertilization with poultry litter in the municipalities of Espírito Santo do Pinhal, Descalvados (C. Camacho, personal communication) and Tambaú (G1 São Carlos e Araraquara, 2019).

In the southern region, there are few records of stable fly outbreaks. In a municipality of Rio Grande do Sul state an outbreak occurred in November (rainy season) 2015 associated with organic fertilization of a pasture with poultry litter in a property with confined and pastured cattle in an integrated system. In the state of Santa Catarina, an outbreak (2018/2019) was attributed to storage of organic fertilizers by the producing company itself (Marvadeza, 2019).

### Contextualized retrospective analysis (1971-2020)

In parallel with the increased frequency of outbreaks associated with sugarcane mills in the last decade, a similar trend, albeit on a smaller scale, was observed in relation to outbreaks associated with other production systems. Here, outbreaks associated with the use of organic fertilizers in several crops are highlighted.

The use of poultry litter and residues/by-products from agricultural production systems as organic fertilizers in several crops is a common practice throughout the country. Records of stable fly outbreaks associated with this practice were rare until the year 2000, with few records found for the previous 30 years. In the following decade (2001-2010), such records increased substantially facilitated by the internet. However, it was just in the second half of the following decade (2011-2020) that a sudden increase in the number of outbreaks became evident, with records of 58 cases in this period, 34 of which in the last three years (2018-2020). This increase, widely publicized in the digital media, was observed mainly in states of the Northeast region, resulting from the use of natural fertilizers.

Poultry litter, commonly known in Brazil as “cama de frango” constitutes the main organic fertilizer associated with stable fly outbreaks in several regions of the country. In general, litter consists of aviary bedding, bird waste, dead skins, feed waste, water, feathers and the associated microbiota (Dalólio et al., 2017). The aviary bedding is the plant material used to cover the floor of the poultry facilities, and usually consist of sawdust, wood shavings, husks (rice, peanuts, coffee and beans), mulch (rice, wheat, barley, rye, corn, beans, soybeans, etc.), grass hay, cassava branches, crushed corn cobs and residues from the sugarcane industry, among other materials (Ávila et al., 1992). Stable fly outbreaks associated with this practice tend to have a yearly frequency due to the systematic use of poultry litter at planting time.

The manure of laying hens is generally richer in nitrogen, calcium and phosphorus as a function of their diet (Figueroa, 2008; Fukayama, 2008). In facilities with less technology, this manure accumulates under the cages and is collected manually, while in more technical production systems, litter management is automated. In either case, the manure must be composted, anaerobically biodigested or biomass burned before being used in agriculture (Augusto & Kunz, 2011).

Outbreaks have occurred yearly in several municipalities in southern Bahia due to fertilization with poultry litter at the time of coffee harvest and papaya planting (Gomes, 2020). Organic fertilizers such as poultry litter and coffee mulch have been used in the cultivation of papaya, banana and coffee for more than a decade in that state (Sulbahia News, 2009; Gomes, 2020). The severity of the outbreaks in the state of Bahia led to the regulation of transport and use of poultry litter in the state by the Portaria 146 from June 7, 2013 (Bahia, 2013) from the State Agency for Agricultural Defense of Bahia (ADAB). As a consequence of the outbreaks, the municipality of Presidente Tancredo Neves, in the state of Bahia, instituted their own regulation through the Municipality Law 261 from December 31, 2013 (Bahia, 2014) requiring technical monitoring of handling and application of such fertilizers in the field and establishing a closed season (May to August) for transport and use of poultry litter.

In the transition between Mata and Agreste mesoregions of the state of Pernambuco, yearly stable fly outbreaks have occurred for more than eight years due to the use of poultry litter as a fertilizer in yam plantations (Redação Nordeste Rural, 2015, 2019) and still continue to occur every year. The frequency and intensity of these outbreaks led the Pernambuco Agricultural Defense and Inspection Agency (ADAGRO) to create the Portaria 31 from May 14, 2014 (Pernambuco, 2014), that regulated the transport and use of organic material from poultry farms in the state in order to prevent the excessive proliferation of stable flies in the vicinity of the crops, as well as to protect the health of livestock and the population from the attack of this pest. The continuity of the outbreaks affecting livestock culminated in a Term of Adjustment of Conduct (TAC) of local farmers to regularize the management of poultry litter use as fertilizer (Pernambuco, 2016).

Stable fly outbreaks resulting from the use of organic fertilizers without adequate prior processing have also been reported in other states, in distinct production systems, such as due to the use of poultry litter in banana plantations in the state of Maranhão (L. M. Costa Júnior, personal communication), orange groves in São Paulo state (ExpressoMT, 2012) and coffee farms and cattle pastures also in the state of São Paulo (C. Camacho, personal communication). A similar situation was reported in a confined animal operation on a farm in the state of Rio Grande do Sul using an integrated system, after a pasture was fertilized with poultry litter, which was used due to the high price of chemical fertilizers. Yearly outbreaks between December and February in a coffee growing region of the state of Minas Gerais were caused by fertilization of coffee plantations with residues from coffee production and processing, a situation aggravated by the rains and eventually by the addition of swine slurry. In sporadic situations like these, regulatory actions rarely occur.

Since the 1970s, outbreaks of stable flies due to the use of fertilizers, particularly coffee wastes, have occurred in municipalities of the state of Espírito Santo. With its economy based on the cultivation of bananas and dairy farming, the municipality of Alfredo Chaves (Espírito Santo state) had serious problems in the 1990s due to the use of coffee mulch in banana cultivation, with severe consequences for dairy production (C. Fanton, personal communication). Recently, in 2020, an outbreak occurred in the municipality of Linhares (Espírito Santo state) caused by the use of poultry litter on papaya (C. Fanton, personal communication). The severity of the socioeconomic problems from those outbreaks culminated in the mandatory control of this pest and the prohibition of the use of decomposing organic substrates without the adoption of stable fly control measures in the state, as established by Portaria 23-R of December 2, 2003 (Espírito Santo, 2003). The aforementioned ordinance also mandated a broad and permanent action by the Capixaba Institute for Research, Technical Assistance and Rural Extension (INCAPER) in the dissemination of methods for controlling that fly.

Another by-product occasionally used as an organic fertilizer is the soy hull - the layer that covers the grain, resulting from the industrial processing of soybeans for oil extraction. In Buritizal (São Paulo state), large numbers of stable fly larvae and pupae were found on a coffee plantation fertilized with this material in a farm near an ethanol mill (T. F. de Souza, personal communication). The proximity between agricultural systems with a high capacity of stable fly production favors a potential interaction and massive production of flies.

The increase in agricultural and poultry production in Brazil, whether due to increased production capacity or the expansion of cultivated areas, has contributed to greater production and use of organic fertilizers. With a national production of poultry litter estimated at 8-10 million tons per year, the main destination of this residue is land application as organic fertilizer and/or in the manufacture of organo-mineral fertilizers (Dalólio et al., 2017). Although the low cost, and ease of use, of this substrate is advantageous for farmers, its high attractiveness and suitability for fly reproduction represent a risk for stable fly outbreaks.

## Outbreaks Associated with Livestock and Integrated Crop-livestock Systems

This category includes outbreaks that originate from common livestock production systems or from hybrid systems, such as integrated crop-livestock systems or integrated crop-livestock-forest systems. Integrated crop-livestock systems are ecologically based farming systems for production of grains, fibers, meat, milk and wool in the same area, carried out in a simultaneous, sequential or rotational schedule (Macedo, 2009; Bonaudo et al., 2014).

High stable fly infestations on cattle in livestock facilities may result from the accumulation of leftover feed, silage and hay, commonly mixed with animal wastes (feces and urine). The disposal or accumulation of these substrates in open areas, without proper treatment or protection against rain, favors their fermentation and attractiveness to stable flies for egg laying and subsequent development of large numbers of larvae.

Even in non-integrated systems, stable fly outbreaks may occur due to the improper management of feed and wastes, such as in feedlots and semi-confinements. In some regions of the United States, stable fly outbreaks associated with hay bales provided to cattle are a yearly problem; once spread on the soil, the fermenting hay mixed with cattle manure and urine can produce up to 19,600 flies/m^2^ (Broce et al., 2005). Outbreaks related to the use of hay have been reported in some Brazilian states as well, mainly in the rainy season.

Outbreaks in integrated crop-livestock systems usually occur in farms with crop (soybean) production and intensive fattening of pastured cattle, although other crops (corn, sorghum, cotton) and feedlots may be present. The dynamics of stable fly outbreaks in integrated crop-livestock systems involve the successive accumulation of mulch on the ground from the no-tillage system of crop production coupled with low-density grazing. The addition of cattle wastes to the organic layer already present on the ground as well as the high heat and humidity due to rains offer a favorable condition for the massive development of larvae and the consequent explosion of stable fly populations. Regardless of its development in pasture mulch with animal wastes, it is suspected that the stable fly can also develop in post-harvest fermented soybean residues. Occasionally stable fly outbreaks have also been associated with slurry from swine farms used as a fertilizer in pastures, as reported in municipalities from Mato Grosso and Minas Gerais.

### Chronological recording

No records of stable fly outbreaks related to livestock practices (other than the use of fertilizers) or integrated crop-livestock systems were found before 2011, when an outbreak in pastured beef cattle, attributed to the development of stable flies in wet spots of a soybean plantation, occurred on an integrated crop-livestock farm with confinement in the state of Mato Grosso do Sul.

Information on outbreaks in the North region has been quite rare, with only two episodes, in the states of Pará and Tocantins, reported by local veterinarians. In both cases, no relationship with organic fertilizers or ethanol mills was plausible. In the episode in Pará (2017), 2,500 insecticide ear tags were unsuccessfully applied in an attempt to contain the outbreak in pastured beef cattle, thus confirming the inefficiency of this strategy in controlling *S. calcitrans* (Guglielmone et al., 2004). The outbreak occurred on the beef cattle ranch in Tocantins (2019) was attributed to development of larvae in a large amount of accumulated feed wastes spilled from troughs and in a thick layer of forage mulch in the pasture during a rainy period, ultimately leading to cattle bunching.

In the Midwest region, 16 outbreaks were reported from 2011 to 2020 in integrated crop-livestock systems in the states of Goiás (4), Mato Grosso do Sul (5) and Mato Grosso (7). Two of the outbreaks in Goiás occurred in consecutive years (May-June 2019/2020) on the same farm, with beef cattle bunching near irrigated pastures and irrigated beans planted after the soybean harvest; according to the farm veterinarian, the outbreaks began and ended with the bean cultivation, leading to the suspicion that the outbreaks were related to mulch in the pasture or soybean residues under the beans. In at least two outbreaks occurring in Mato Grosso do Sul state, local producers or veterinarians observed low-density grazing and pasture mulch on the ground in farms with integrated crop-livestock systems. A similar situation, also associated with an integrated system, was reported in 2019 on a farm located in Salto del Guairá (Paraguay), close to the Brazilian border south of Mato Grosso do Sul state (S. Kassab, personal communication).

Outbreaks with similar characteristics were reported from 2016 to 2019 in at least seven municipalities of the state of Mato Grosso. All of them occurred during the rainy season (mainly January/February) on large integrated crop-livestock farms and confinement and/or semi-confinement livestock facilities. Other contributing factors were accumulations of unharvested soybean residues and the feeding of a large amount of hay (2,000 bales) in the dry season prior to the outbreak.

Severe outbreaks in pastured beef cattle during the rainy seasons of 2017 and 2018 occurred on an integrated crop-livestock farm in Bahia state involving confinement, low-density grazing, accumulation of mulch (at least 15 cm from several crops) on the ground, wet silage, and heavy rains. According to the veterinary owner, in the 2017 outbreak, losses in weight gain due to stable fly infestations were estimated at around 45 tons and in 2018 at least five bovine deaths were recorded on the farm.

### Contextualized retrospective analysis (1971-2020)

Proximity tends to be a key factor in the occurrence of outbreaks involving agricultural and livestock production systems since it greatly facilitates the movement of flies between feeding (livestock) and immature developmental (crop areas) sites. In integrated production systems, the ease tends to be even greater since livestock and crops may be adjacent (if simultaneous) or even in the same place (if alternated). Stable fly outbreaks of this nature have been reported mainly in the Midwest, including the states of Goiás, Mato Grosso do Sul e Mato Grosso, often associated with cattle raising and crops such as soybeans.

The accumulation of cultural residues from crops on the ground creates a more humid and more temperate microclimate, making it a favorable environment for the development of fungi, insects and other invertebrates that feed on organic matter, particularly in the rainy season (Fernandes & de Oliveira, 1997; Oliveira et al., 2009). With a greater prevalence and diversity of arthropods in no-tillage systems, such approaches may demand the adoption of adequate pest management strategies (House & Stinner, 1983).

First recorded in the last decade, stable fly outbreaks associated with integrated crop-livestock systems have become an emerging problem in some states, with a noticeable increase in the number of cases over the last few years (Table 1). As a relatively small-range problem, with both origin and damage generally occurring in the same farm, such outbreaks do not result in complaints or conflicts among production chains, and therefore are rarely reported by the media. In fact, all of the records presented here came from personal contacts with producers or technicians seeking guidance. The absence of information from the media clearly indicates that these cases do not represent the actual magnitude of this problem.

Due to the lower availability of substrates for larval development throughout the year (in terms of both area and availability over time), outbreaks associated with integrated crop-livestock systems tend to have lower intensity, duration and scope than those associated with ethanol mills. On the other hand, the growing adoption of integrated systems in the Brazilian Midwest suggests that the frequency of stable fly outbreaks tends to increase in this production system, as observed in recent years. Thus, the association between outbreaks and integrated systems demands attention and the adoption of efficient preventive measures, so that the expansion of this production system does not result in the parallel expansion of the stable fly problem.

## Mapping and Spatial Distribution of Outbreaks

The close association between stable fly outbreaks and production systems shows relatively well-defined trends and/or regional patterns in terms of their geographic occurrence. Ultimately, these trends depend on the regional distribution of crops, their management, and the residues/by-products generated, stored and/or used in each productive activity.

**Figure 2:**
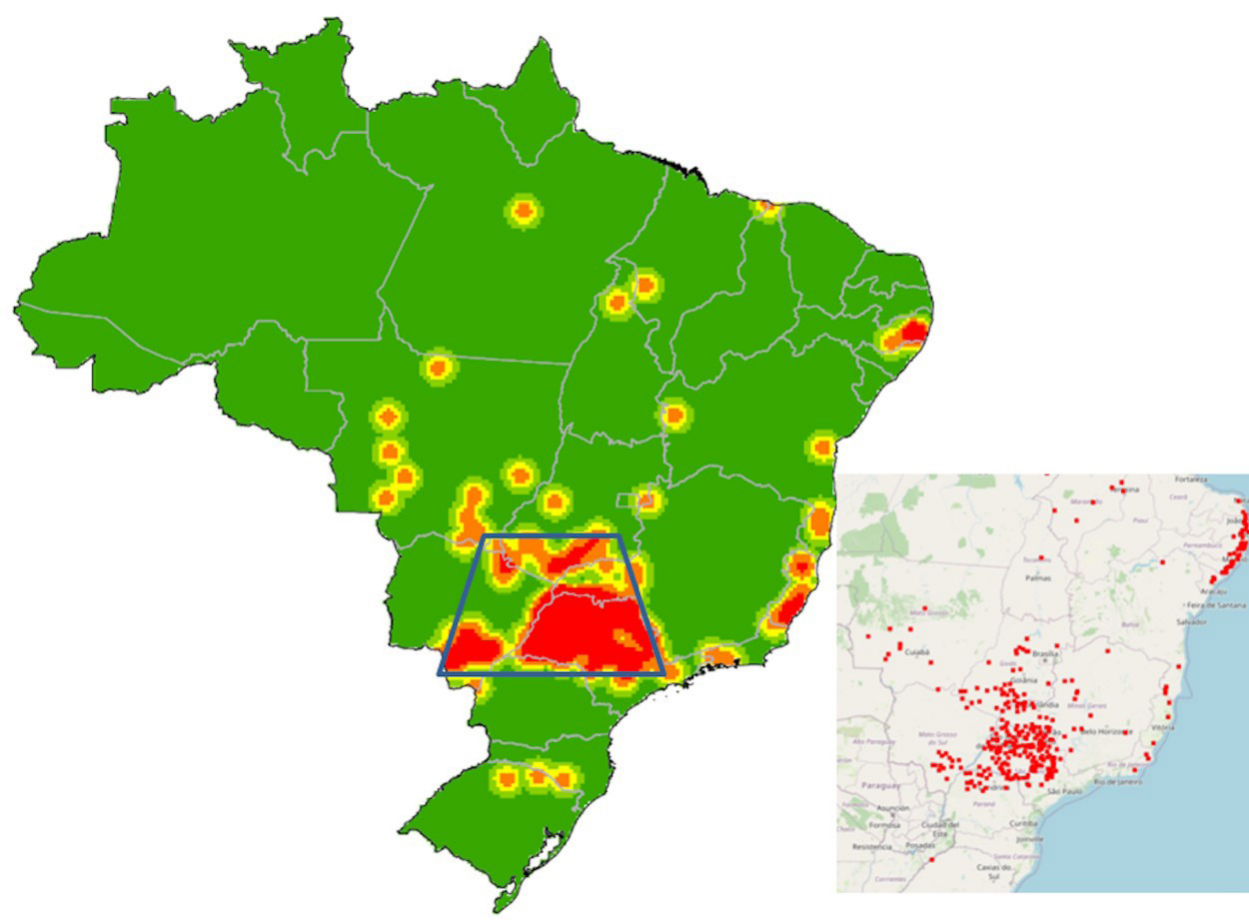
Stable fly outbreaks in Brazil from 1971 to 2020 (large map) and current distribution of sugarcane ethanol mills (small map; CONAB, 2021).

Municipalities affected by stable fly outbreaks are concentrated in the southeast and midwest regions of the country (Figure 2) and tend to be associated with sugarcane mills. This distribution can be represented by a polygon covering the states of São Paulo (mid and northwest), Mato Grosso do Sul (south), Minas Gerais (west), Mato Grosso (south) and Goiás (south), which coincides with the distribution of ethanol mills in the referred regions (Figure 2).

The absence of outbreaks of the stable fly associated with sugarcane mills in the Northeast region can be explained by the pre-harvest burning of sugarcane, which is allowed and performed routinely. Economic, administrative and operational issues hinder the continuous adoption of efficient, preventive management measures by the sugarcane mills, which helps to explain the recurrence of the problem in several municipalities.

Since the use of organic fertilizers in several crops is a common practice throughout the country, outbreaks resulting from this source are more dispersed (Table 2). Despite this, regional patterns are observed and depend on the location of production chains or production systems, as well as the availability and/or ease of obtaining organic fertilizers.

Applied to different crops, poultry litter has caused outbreaks in several states, including Bahia, Pernambuco, Maranhão, São Paulo and Rio Grande do Sul. In the Northeast, outbreaks have been recurrent due to its use in the production of fruits, mainly banana (Bahia and Maranhão) and papaya (Bahia), as well as in the production of tubers, such as yam (Pernambuco). Outbreaks with this origin have been sporadic in other states, such as in RS, resulting from the fertilization of pasture with poultry litter, and in São Paulo state, associated with the use of this fertilizer in coffee and orange plantations.

Coffee mulch is another fertilizer associated with the occurrence of stable fly outbreaks. In general, the geographic distribution of these outbreaks is associated with some of the main coffee regions in the country, occurring mainly in the state of Espírito Santo, but also in Bahia (south) and Minas Gerais states. This is the main cause of more than 80% of the outbreaks reported in Espírito Santo (Table 2). Although a few outbreaks in the states of Rio de Janeiro and Espírito Santo were related to the use of organic fertilizers, the much higher frequency of outbreaks in the state of São Paulo associated with the sugarcane activity makes this origin the most important regionally (Table 2).

Outbreaks associated with livestock and integrated crop-livestock production systems have occurred mainly in states of the Midwest region. Extensive livestock farming is widely carried out in the region and the adoption of integrated systems has expanded, enabling wider use of the property in the crop off-season periods. Outbreaks associated with integrated crop-livestock systems have been reported mostly in the states of Mato Grosso, Mato Grosso do Sul and Goiás, but there are also records in Bahia (Table 2).

## General Outlook and Final Comments

The production records resulting from the intensification and expansion of agricultural, livestock and agro-industrial production systems over the last few decades represent a remarkable achievement of national agribusiness. However, both the expansion and management changes, along with the interaction (purposeful or not) between production systems, may eventually favor the abundance of pest species that benefit from greater ease and availability of food and developmental habitats. This seems to be the case behind the population explosions of stable flies, an insect which has shown a remarkable ability to adapt and use different resources for its development.

**Figure 3:**
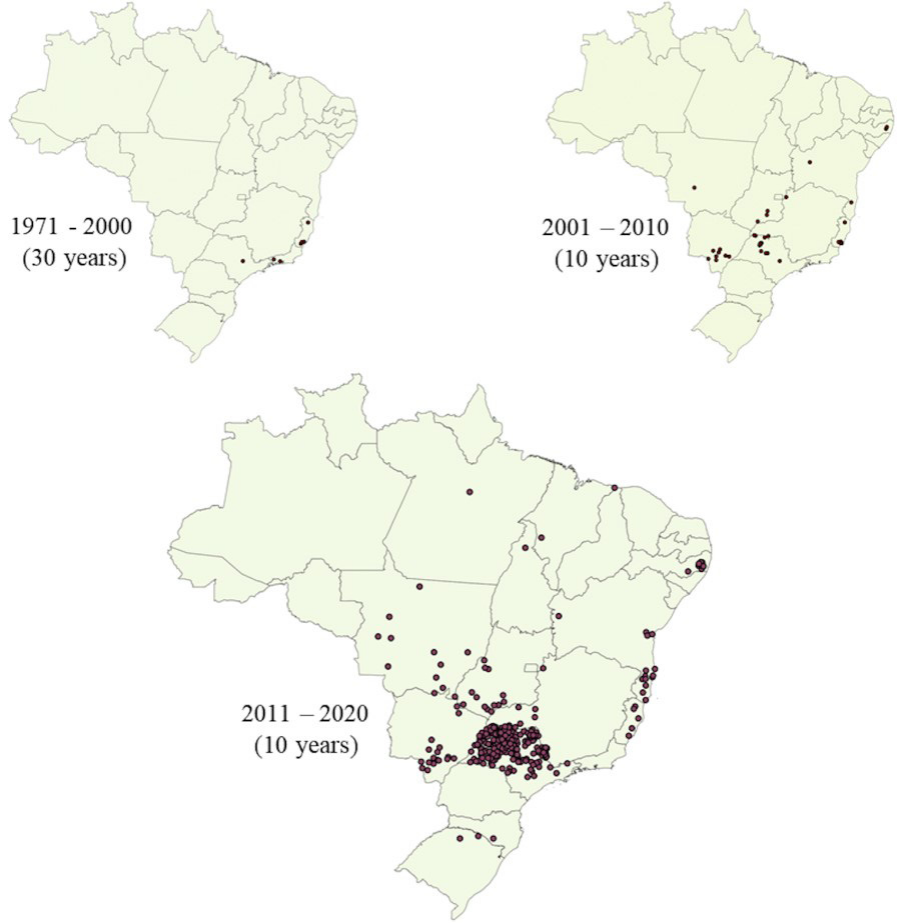
Evolution of stable fly outbreaks recorded in Brazil in five decades (1971 to 2020).

In the last five decades, a huge increase in the frequency of stable fly outbreaks has been registered in the country (Figure 3). Obviously, the number of records currently available would not be the same without the internet, but the increase in the number of outbreak cases was evident over the years driven by the expansion of production chains, increase in the production scale and changes in management, some of which are due to legal decisions. Due to their outstanding importance, two sources of outbreaks - by-products from ethanol mills and organic fertilizers - deserve specific comments regarding some macro trends.

Projections and scenarios that supported the National Energy Plan 2050 foresee an increase in demand for electricity and ethanol, pointing to an expansion of the Sugar-Alcohol/Bioenergy Sector in the coming decades (Brasil, 2018). Around 80% of the world's ethanol production is currently supplied by Brazil and the United States, but Brazil is able to sustainably expand its participation in the domestic and international market (Brasil, 2020). Thus, the global trend of replacing fossil fuels with more sustainable alternatives and the growing demands for domestic consumption and exports of ethanol presuppose continuity in the expansion of the sector, with a recent history of expansion toward the Midwest.

The expansion of the bioenergetics sector in areas occupied by livestock favored the use of both productive environments by the stable fly, predisposing an increase of outbreaks, as observed in the Midwest in the last decade. As long as the current use of technologies, scale of production and generation of residues and by-products of the ethanol industry persist, it would be advisable to adopt management techniques that curtail risks of this production system to other production chains, the environment and the surrounding populations. Although substrates such as filter cake and vinasse sludge can also trigger outbreaks, their scale of production is not equal to that of fertigation in sugarcane fields. Thus, in the dynamics of outbreaks associated with ethanol mills, the mulch-vinasse blend is the main inducer of stable fly outbreaks, deserving special attention and care.

The application of vinasse requires specific management for prevention of stable fly outbreaks. The volume of vinasse to be applied should not be determined just by the potassium content present in the soil, as is currently done for optimum sugarcane production, but also by the moisture left on the mulch deposited on the ground, since it is a key factor for the development of immature stable flies.

Far from being a simple matter, moisture management is influenced by several operational and abiotic factors. Hopefully, new technologies may bring useful advances to this challenge. The generation of energy from sugarcane mulch, as well as other technologies, may become a valuable approach to support the growth in demand for electricity and the need for a greater supply of renewable energy inputs; however, it depends on the preservation of agronomic conditions and competitive costs (Brasil, 2020).

The expansion of national poultry production in recent decades has allowed Brazil to reach the honorable position of the third largest producer of chicken meat in the world (AviSite, 2021). However, the intensification of poultry production directly results in greater production of wastes and, indirectly, in its increasing use as a fertilizer in crops. The maintenance of high poultry productivity and the expansion of Brazilian agribusiness, which includes the use of *in natura* poultry litter as the main organic fertilizer in the country, tends to increase the risks of stable fly outbreaks (also house flies) as has happened in the last decade.

Regardless of the crop, the use of organic fertilizers requires the adoption of preventive measures. Fertilizer treatment (as composting) and/or incorporation into the soil reduce the risks associated with attractiveness to flies and favorability for larval development, which ultimately reduces subsequent outbreaks. Stable fly outbreaks due to poultry litter involve three production chains (i.e., poultry, crop, livestock), and specific legislation has focused on fertilizer transport and the end user (farmer) in states where such outbreaks have become a serious problem. Such legislation has caused conflict between parties involved and has had a somewhat limited efficiency in reducing cases. The proper processing of this fertilizer at its origin, before commercialization, may reduce risks of fly outbreaks as well as add value to the commercialized products.

Adequate care of organic residues and by-products, the judicious use of fertilizers (whether vinasse or poultry litter) and the adoption of management practices to avoid the establishment of large fly developmental sites should be a goal in all production systems associated with stable fly outbreaks. Ultimately, such measures will reduce problems and losses to all the production systems involved, as well as to the environment.

The search for processes that have a preventive effect on the occurrence of outbreaks is especially important, since current measures for controlling stable fly outbreaks affecting cattle are few and inefficient. Several commercially available insecticidal products are highly effective against stable fly larvae and adults; however, their use is limited by legal issues. The use of products such as larvicides is restricted by the lack of registration or permission for their use in different crops, including sugarcane. Regarding the chemical control of adult flies, the application of insecticides on animals is quite inefficient (quickly removed from legs by the pasture) and their application in refuge areas of the adult fly, such as reserves and environmentally protected areas, is obviously prohibited. Thus, preventive actions are the most efficient way to reduce the occurrence and mitigate the intensity of these outbreaks.

The explosion of stable fly outbreaks during the 2010s gave rise to several studies aimed at understanding the dynamics of the problem, as well as subsidizing preventive and corrective technical recommendations. Given the frequency and severity of the outbreaks as well as the importance of the bioenergy sector in the national context, those studies have focused almost exclusively on outbreaks associated with the ethanol mills. Such studies demonstrated the association between outbreaks and sugarcane mills (Bittencourt, 2012; Kassab et al., 2012), the high potential of stable fly production in ethanol by-products (Corrêa et al., 2013), the dynamics of outbreaks and relationship with the sugarcane management and the off-season maintenance of stable fly populations in livestock farms (Dominghetti, 2017), and the attractiveness of vinasse to stable flies (Souza et al., 2021). Similar studies should be expanded to other production systems associated with outbreak episodes, mainly those using organic fertilizers and integrated crop-livestock.

The information presented in this survey provides an unprecedented picture of the occurrence of stable fly outbreaks in the country over the last 50 years. More than a collection of records, this publication presents a history of the evolution of stable fly outbreaks in Brazil, their association with different production systems, geographic distribution and relationship with legal resolutions. It is hoped that such information may support actions and decisions aimed at preventing this problem and mitigating its serious consequences for the affected human and animal populations, as well as the environment.
